# Author Correction: Ancient genomes reveal social and genetic structure of Late Neolithic Switzerland

**DOI:** 10.1038/s41467-020-18561-y

**Published:** 2020-09-16

**Authors:** Anja Furtwängler, A. B. Rohrlach, Thiseas C. Lamnidis, Luka Papac, Gunnar U. Neumann, Inga Siebke, Ella Reiter, Noah Steuri, Jürgen Hald, Anthony Denaire, Bernadette Schnitzler, Joachim Wahl, Marianne Ramstein, Verena J. Schuenemann, Philipp W. Stockhammer, Albert Hafner, Sandra Lösch, Wolfgang Haak, Stephan Schiffels, Johannes Krause

**Affiliations:** 1 Institute for Archaeological Sciences, Archaeo- and Palaeogenetics, University of Tübingen, Tübingen, Germany; 2 Max Planck Institute for the Science of Human History, Jena, Germany; 3 ARC Centre of Excellence for Mathematical and Statistical Frontiers, School of Mathematical Sciences, The University of Adelaide, Adelaide, SA 5005 Australia; 4 Department of Physical Anthropology, Institute of Forensic Medicine, University of Bern, Bern, Switzerland; 5 Institute of Archaeological Sciences, Prehistoric Archaeology, University of Bern, Bern, Switzerland; 6 Archaeological Office of the District of Constance, Konstanz, Germany; 7 Department of history of arts and Archaeology, University of Burgundy, Burgundy, France; 8 Museum of Archaeology Strasbourg, Strasbourg, France; 9 Institute for Archaeological Science, Palaeoanthropology, Eberhard Karls University Tübingen, Tübingen, Germany; 10 State Office for Cultural Heritage Management Baden-Wuerttemberg, Konstanz, Germany; 11 Archaeological Service of the canton of Bern, Bern, Switzerland; 12 Senckenberg Centre for Human Evolution and Palaeoenvironment, University of Tübingen, Tübingen, Germany; 13 Institute of Evolutionary Medicine, University of Zurich, Zurich, Switzerland; 14 Institut für Vor- und Frühgeschichtliche Archäologie und Provinzialrömische Archäologie, Ludwig Maximilian University, Munich, Germany; 15 Oeschger Centre for Climate Change Research, University of Bern, Bern, Switzerland

**Keywords:** Evolutionary genetics, Population genetics, History

Correction to: *Nature Communications* 10.1038/s41467-020-15560-x, published online 20 April 2020.

The original version of this article contained several errors, listed below, resulting from an update to the Y chromosomal haplogroup assignments during the final revisions. These do not affect the results or conclusions of the work.

The original version of this article contained an error in Fig. 4, several labels of ‘Y na’ have now been replaced by ‘Y’ in the corrected figure, the colour of ‘mt’ has also been changed in the corrected figure. The correct version of Fig. 4 is below:  which replaces the previous incorrect version: This has been corrected in both the PDF and HTML versions of the article.

The original version of the Supplementary Information associated with this article contained an error in Supplementary Note 4, line 9, which incorrectly read ‘In the younger site of Spreitenbach associated with the Corded Ware complex only haplogroups of the clades I2c and I2a are present’. The correct version states ‘In the younger site of Spreitenbach associated with the Corded Ware complex only haplogroups of the clade I2a is present’. It also contained an error in Supplementary Note 4, line 11, which incorrectly read ‘All samples in the dataset younger than 2200 BC all individuals carry R1b, beside MX265 from Singen, which belongs to R1a’. The correct version states ‘After 2200 BC all individuals carry R1b, beside MX265 from Singen, which belongs to R1a’.

The original version of the Supplementary Table 5 in the Supplementary Information had several errors in column 16, as well as an error in column 9 (line 15). A line has also been added on line 31. The HTML has been updated to include a corrected version of the Supplementary Information; the original incorrect version can be found as Supplementary Information associated with this Correction.

The original version of the Supplementary Data 1 file contained several errors in columns G, H and X. The HTML has been updated to include a corrected version of Supplementary Data 1; the original incorrect version of Supplementary Data 1 can be found associated with this Correction.

## Supplementary information

Supplementary Information

