# Supplementary material for: Ancient genomes reveal social and genetic structure of Late Neolithic Switzerland
Source: Nat Commun. 2020 Apr 20;11:1915. doi: 10.1038/s41467-020-15560-x (PMC7171184; doi:10.1038/s41467-020-15560-x)
Supplement: Supplementary file 1 — Supplementary Information [file 41467_2020_15560_MOESM1_ESM.pdf]

Table of Contents

Supplementary Note 1: Archaeological information of the sites included in this study 2

Supplementary Note 2: Radiocarbon dating of the newly sequenced individuals 5

Supplementary Note 3: Mitochondrial genome reconstruction... 6

Supplementary Note 4: Y-chromosomal haplogroup assignment 7

Supplementary Note 5: ADMIXTURE Analysis... 10

Supplementary Note 6: Sex-biased Admixture 12

Supplementary Note 7: Proportions of steppe ancestry on individual level 13

Supplementary Note 8: Estimated arrival time of the steppe component in the different regions 15

Supplementary Note 1: Archaeological information of the sites included in this study

<u>Middle Neolithic</u>

CH Niederried Ursisbalm 4458 – 4341 BCE (2sigma cal.)

Tschumi 1920, Hug 1956, Schlaginhaufen 1924

The stone cist burial near Niederried was discovered and excavated in 1913. After rock blasting on a construction site, the graves were found below. The burial had an NW-SE orientation and was constructed with five tiles of chalk. The individual in the grave, an adult, was found in crouched position. Outside the stone cist, the bones of two subadults were found and probably from an earlier burial which was later removed from the cist. Four additional stone cist burials were destroyed by further blasts. Samples from two individuals were used for genetic analyses: A1 and A2 and showed sufficient DNA preservation. The two individuals, one female, and one male are unrelated. Radiocarbon dating hints to an age of 4458 – 4341 calBCE. Samples were provided by the Archaeological Service of the canton of Bern.

F Lingolsheim 4786 – 2208 BC (2sigma cal.)

Lichardus-Itten 1980

The burial ground of Lingolsheim was discovered in spring 1910 in a sandpit and comprises at least 20 graves. The individuals were found in stretched supine position in NW-SE orientation. From these individuals, one petrous bone from tomb 15 was used for genetic analysis. The male individual was dated to 4766-4601 calBCE.

Between 1923 and 1926 a second field session took place in a nearby second sandpit and 17 more graves were discovered. Four of these individuals were buried in crouched position in W-E orientation. Based on found potsherds the graves were assigned to the Rubané culture. From this group, the individual from tomb E was sampled for this study. The male individual dates to 2463-2208 calBCE contradicting the cultural assignment.

The 13 remaining burials were also found in stretched supine position and as the former ones from 1910 assigned to the Grossgartach culture. Petrous bones from the graves 18, 32 and 35 were used for DNA analysis. Only the individuals 18 and 35, a male and a female, contained enough DNA for downstream analysis. Only individual 35 could be dated to 4786-4618 calBCE. Samples were provided by the Museum of Archaeology in Strasbourg.

<u>Late Neolithic</u>

CH Oberbipp 3350-2650 BCE (2sigma cal.)

Ramstein et al. 2014, Siebke et al. 2018, Siebke et al. 2019, Steuri et al. 2019

The Dolmen burial from Oberbipp was excavated in 2012. The site is situated on the Swiss Plateau and on the southern slope of the Jura mountains. The construction of the dolmen consists of large glacial erratics in the form of blocks and slabs. More than 2000 fragmented and commingled skeletal elements were retrieved at the excavation and a minimal number of 42 individuals as determined from femora including males and females of all age classes. An extended supine position could be reconstructed for the buried individuals in the grave chamber. In total 22 petrous bones from different individuals and 46 teeth could be sampled for aDNA analyses and radiocarbon dating. Samples were provided by the Archaeological Service of the canton of Bern.

CH Muttenz 3010 – 2706 BC (2sigma cal.)

Muttenz, and Hagmann D, editors. 2009; Siebke et al. 2019

The multiple burial of Muttenz was discovered in 1946. Details about the finding situation and the excavation are unknown. It contained at least 11 individuals of which five petrous bones from different individuals could be sampled for aDNA and radiocarbon dating. Samples were provided by the Archaeological Service of the canton of Basel Landschaft.

CH Aesch 3094 – 2500 BC (2sigma cal.)
Löhlein 2011; Stöckli 1995, Schegler 2016, Bay 1936
The dolmen burial of Aesch was excavated in 1907 and 1909. The megalithic burial consisted of one rectangular grave chamber 4m long and 3m wide with a flagged floor. Skeletal remains of 33 adult individuals and 14 children were recovered. 25 of the skeletons comprised petrous bones, which were used for DNA analysis and radiocarbon dating. Samples were provided by the Archaeological Service of the canton of Basel Landschaft.

<u>Final Neolithic/ Early Bronze Age</u>
CH Spreitenbach 2900 – 2031 BC (2sigma cal.)
Doppler 2012, Bleuer et al. 1999
In 1997 the multiple burial of Spreitenbach, Canton Aargau in Switzerland was discovered and excavated. The former wooden construction contained five males, four females, two subadults and a newborn in crouched position. The burials are radiocarbon dated to approximately 2500 BCE. All 12 individuals (pars petrosae) could be sampled for additional aDNA analyses. Samples were provided by the Archaeological Service of the canton of Argau.

CH Bad Zurzach 2206 – 1946 BC (2sigma cal.)
Bleuer et al. 2012, Doswald et al. 1989
The double burial of Bad Zurzach was excavated in 1984. The grave-pit is in N-S orientation without further stone or wooden construction and contained two adult individuals in contracted position. A contemporaneous burial of the two male individuals is assumed. For both a mature age of death is estimated. The burial was assigned to the Early Bronze Age and both individuals were sampled for aDNA analyses. Samples were provided by the Archaeological Service of the canton of Argau.

CH Rapperswil 2695 – 2481 BC (2sigma cal.)
Grüninger und Kaufmann 1982
During construction works in 1980, a stone cist burial near Rapperswil was discovered. Within the W-E oriented cist, the skeletal remains of an approximately 35-year-old female buried in extended supine position were found. In the abdominal region, bones of an eight lunar months old and probably unborn foetus were found. From both individuals, mother and child DNA could be retrieved. Samples were provided by the Archaeological Service of the canton of St. Gallen.

CH Wartau 3036 BC – 2 AD (2sigma cal.)
Stehrenberger 2016, Stehrenberger 2019
The human remains are located in a natural cave. In the 1970s and 1980s, several inspections by the archaeological service of the Canton St. Gallen took place. The cave was accessible to the public at all times and in the 1970s some human remains were recovered illegally and were transferred to the archaeological service in 2001. Radiocarbon dates of bone fragments date to the Middle, Late, and Final Neolithic period as well as to the Iron Age/Roman time. Eleven petrous bones were assed for DNA analyses. Samples were provided by the Archaeological Service of the canton of St. Gallen.

D Singen 2199 - 431 BC (2sigma cal.)
Stockhammer et al. 2015, Oelze et al. 2011, Krause 1988
The Early Bronze Age cemetery of Singen (Hohentwiel) was excavated in the 1950s and is one of the largest and most important cemeteries of this period in Southern Germany. In close vicinity, Final Neolithic and later Bronze to Late Iron Age burials were excavated – providing an outstanding continuity of burials at the site. The Early Bronze Age necropolis of Singen is of particular interest, as it was used to define the eponymous “Singen Group” of the Early Bronze Age in Southwestern Germany characterized by the strict positioning of the deceased, stone constructions and particular burial goods. In total, 96 graves were excavated, and it was assumed that they can be related to one kin group. Human remains of approximately 30 individuals were recovered and 15 petrous bones selected for aDNA extraction. Radiocarbon dates of these individuals’ range between 2199 - 431 BC. Samples were provided by the Archaeological Office of the District of Constance.

D Anselfingen 2456-2203 BC (2sigma cal.)
Merkl 2016
The site of Anselfingen “Breite”, Germany was first discovered in 2008 and excavated during several excavation sessions in the years from 2009 till 2017. Multiple Corded Ware and Bell Beaker associated graves could be recovered. Available for DNA analysis was the petrous bone of an adult individual from a double burial associated with the Bell Beaker phenomenon which was excavated in 2010. This individual was buried in N-S orientation and in crouched position and buried with the remains of a small child. Samples were provided by the Archaeological Office of the District of Constance.

CH Auvernier 2866-2601 BC (2sigma cal.)
Schwegler 2016
The dolmen of Auvernier was discovered in 1876 near the lake Neuchâtel. The complex consisted of 12 vertical stone slabs and two horizontal ceiling tiles. In the burial chamber inhumations of 15 to 20 individuals could be found. Marks on the stone slabs indicate the reuse from an older Neolithic construction which was possibly used around 3000 BCE. The rebuilt dolmen is dated at 2800 BCE at the earliest and was used until the Bronze Age. The only DNA could be retrieved from one individual that dates to 2866-2601 calBCE. Samples were provided by the Archaeological Service of the canton of Bern.

CH Burgäschisee 2862-2581 BC (2sigma cal.)
Ulrich-Bochsler 2012; Schlaginhaufen 1924; Wiedmer-Stern 1904
The burial of Burgäschisee was discovered in 1902 near the shore of the lake Burgäschisee. During 1902 and 1943 some human remains were found. In total inhumation of possibly three individuals could be recovered. After the original description, one individual belongs to the Neolithic period and was buried in crouched position. Not all bones of the skeletons were recovered and preserved. The sample of a femur fragment did not provided enough DNA for subsequent analyses and was excluded from population genetic analysis. Samples were provided by the Archaeological Service of the canton of Bern.

CH Seengen 2463-2274 BC (2sigma cal.)
Bleuer et al. 2012
The remains of a stone cist by Seengen was excavated in 1993. The burial contained two individuals, one of them consisted only of cremated remains. The grave structure is archaeologically dated to the Bronze Age. A petrous bone of the unburned human was used for DNA analyses. . Samples were provided by the Archaeological Service of the canton of Argau.

Supplementary Note 2: Radiocarbon dating of the newly sequenced individuals

Every individual sequenced in this study was radiocarbon dated (SI table 1). For the individuals 1, 6/14, 7/11, 9, 10/16 and 13/18 the C^{14} dates from the original publication were adopted (**Doppler 2012**). Samples for the new radiocarbon dates were always taken from the same skeletal element (petrous bone, femur or tooth) from which the bone powder for DNA extraction was taken.
The dating was conducted in the LARA (Laboratory for the Analysis of Radiocarbon with AMS) at the University of Bern. The individuals from Oberbipp were also dated at the Curt-Engelhorn-Zentrum in Manheim. In case of large differences between the results of the two measurements, samples were dated a third time at the ORAU (Oxford Radiocarbon Accelerator Unit) in Oxford (**Steuri et al. 2019**).

All burials are far from coastal areas, therefore the reservoir effect due to the consumption of marine fish can be neglected since reservoir effects in freshwater lakes are not detectable so far. Furthermore, the studied populations were retrieved from burials offsite lake shores. Even though many Neolithic settlements in Switzerland were found at the lake shores, the burials are usually not associated with or in close proximity to them. In addition, the data collected for these settlements indicates a high proportion of husbandry and only limited fish consumption. This is also supported by stable isotope analysis of the studied individuals (**Siebke et al 2019**). Therefore, any possible bias based on “hard water effect” can be neglected as well.

Supplementary Note 3: Mitochondrial genome reconstruction

All libraries were enriched for mtDNA following the procedure described in **Futwängler et al. 2018** and sequenced on an Illumina HiSeq at the Max Planck Institute for the Science of Human History in Jena. After processing the raw reads with EAGER (**Peltzer et al 2016**) contamination estimates and consensus sequences were computed with *schmutzi* (**Renaud et al. 2015**). Shared haplotypes were identified using MEGA-X and haplogroups were determined using *haplogrep* (**Weissensteiner et al. 2016**, SI Tab. 1). Percentual portions of the makrohaplogroups N1a, W, X, H, T2, J, U2, U3, U4, U5a, U5b, K, and U8 were calculated for each site (SI Fig. 1).

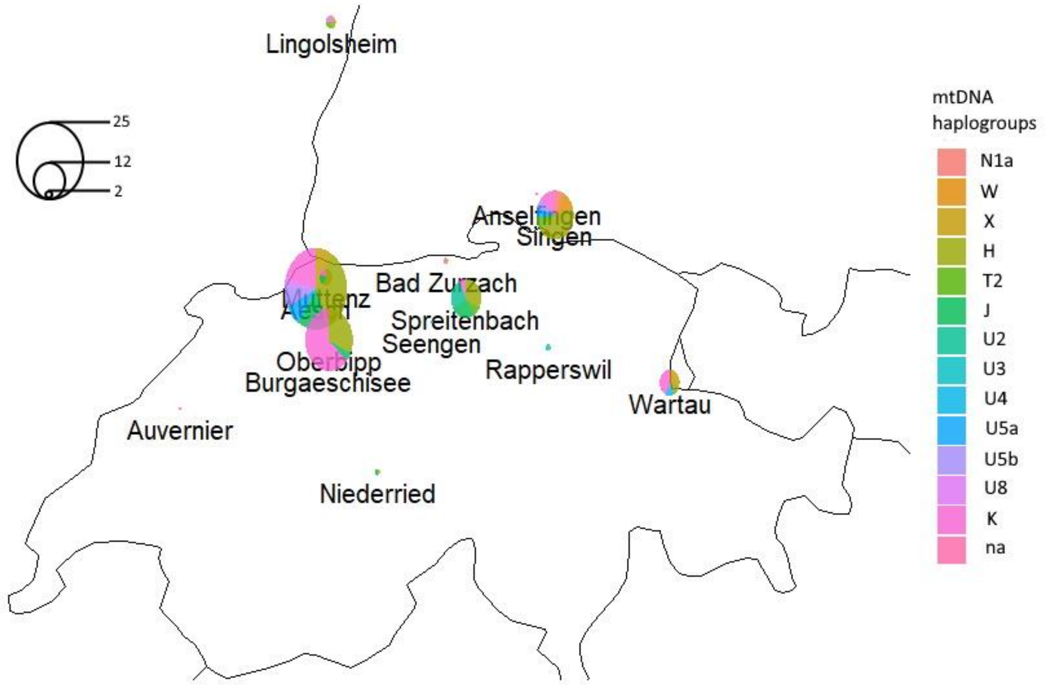

Supplementary Figure 1 | Composition of the mtDNA haplogroups N1a, W, X, H, T2, J, U2, U,3 U4, U5a, U5b and U8 for each site. The size of the circles indicates the sample size for each site. Map generated with R version 3.4.3 (R Core Team 2017) using the CIA World Data Bank II is currently (mid-2003) available from http://www.evl.uic.edu/pape/data/WDB/

Supplementary Note 4: Y-chromosomal haplogroup assignment

Assignment for Y-chromosomal haplogroups was performed via visual inspection of the captured SNPs, following the nomenclature of the International Society of Genetic Genealogy (ISOGG) version 14.218 (retrieved 19 November 2019, http://www.isogg.org).
In individuals older than 2600 BC originating from the sites of Oberbipp, Aesch and Muttenz only haplogroups belonging to the clades I2a and G2a were found. Besides the youngest Aesch individual Aesch25, which carries a R1b haplogroup.
In the younger site of Spreitenbach associated with the Corded Ware complex only haplogroups of the clades I2c and I2a are present. In the contemporaneous sample from Lingolsheim R1b could be determined.
All samples in the dataset younger than 2200 BC all individuals carry R1b, beside MX265 from Singen, which belongs to R1a.

Supplementary Table 1 | Y chromosomal haplogroup assignment for all male individuals

ID	Site	Cal 2 sigma BC	Terminal derived mutation	YHG
Aesch1	Aesch (CH)	3090-2917	PF3239	G2a2a1a2a1
Aesch12	Aesch (CH)	3010-2884	PF3239	G2a2a1a2a1
Aesch13	Aesch (CH)	3016-2901	PF3239	G2a2a1a2a1
Aesch14	Aesch (CH)	3014-2898	PF3239	G2a2a1a2a1
Aesch17	Aesch (CH)	3011-2889	PF3239	G2a2a1a2a1
Aesch19	Aesch (CH)	no collagen	PF3239	G2a2a1a2a1
Aesch20	Aesch (CH)	2913-2878	FGC7739/Z6488	G2a2a1a2a
Aesch21	Aesch (CH)	no collagen	PF3239	G2a2a1a2a1
Aesch22	Aesch (CH)	2892-2694	PF3239	G2a2a1a2a1
Aesch23	Aesch (CH)	2881-2676	PF3239	G2a2a1a2a1
Aesch24	Aesch (CH)	2912-2877	FGC7739/Z6488	G2a2a1a2a
Aesch25	Aesch (CH)	2864-2501	L51/M412/PF6536/S1	R1b1a1b1a1a
Aesch4	Aesch (CH)	3094-2926	PF3239	G2a2a1a2a1
Aesch6	Aesch (CH)	2905-2759	PF3147	G2a2a
Aesch7	Aesch (CH)	no collagen	FGC7739/Z6488	G2a2a1a2a
MX150	Oberbipp Horgen (CH)	3244-3102	L91/PF3246/S285	G2a2a1a2
MX182	Oberbipp Horgen (CH)	3338-3031	PF3239	G2a2a1a2a1
MX183	Oberbipp Horgen (CH)	3344-3037	FGC7739/Z6488	G2a2a1a2a
MX187	Oberbipp Horgen (CH)	3337-2908	PF3239	G2a2a1a2a1
MX188	Spreitenbach CWC (CH)	2495-2399	M423	I2a1a2
MX190	Spreitenbach CWC (CH)	2860 - 2460	M423	I2a1a2
MX191	Spreitenbach CWC (CH)	2570 - 2190	M423	I2a1a2
MX192	Spreitenbach CWC (CH)	2571-2513	M423	I2a1a2
MX195	Spreitenbach CWC (CH)	2470 - 2050	M423	I2a1a2
MX204	Oberbipp Horgen (CH)	no collagen	FGC7739/Z6488	G2a2a1a2a
MX209	Oberbipp Horgen (CH)	no collagen	PF3239	G2a2a1a2a1

ID	Site	Cal 2 sigma BC		YHG
MX210	Oberbipp Horgen (CH)	no collagen	PF3239	G2a2a1a2a1
MX211	Oberbipp Horgen (CH)	3100-2928	PF3239	G2a2a1a2a1
MX212	Oberbipp Horgen (CH)	3323-2581	PF3239	G2a2a1a2a1
MX213	Oberbipp Horgen (CH)	3363-2930	PF3239	G2a2a1a2a1
MX219	Oberbipp Horgen (CH)	3330-3216	PF3147	G2a2a
MX252	Singen (D)	1941-1774	L2/S139	R1b1a1b1a1a2b1
MX254	Singen (D)	no collagen	L2/S139	R1b1a1b1a1a2b1
MX257	Singen (D)	1879-1696	L2/S139	R1b1a1b1a1a2b1
MX258	Singen (D)	2028-1903	P312/PF6547/S116	R1b1a1b1a1a2
MX259	Anselfingen (D)	2456-2203	P312/PF6547/S116	R1b1a1b1a1a2
MX265	Singen (D)	763-431	L146/M420/PF6229	R1a
MX270	Singen (D)	no collagen	L2/S139	R1b1a1b1a1a2b1
MX275	Singen (D)	2135-1961	L2/S139	R1b1a1b1a1a2b1
MX279	Singen (D)	1882-1745	L2/S139	R1b1a1b1a1a2b1
MX283	Singen (D)	2116-1926	L2/S139	R1b1a1b1a1a2b1
MX286	Singen (D)	2029-1892	L2/S139	R1b1a1b1a1a2b1
MX288	Singen (D)	2199-2028	L2/S139	R1b1a1b1a1a2b1
MX298	Wartau (CH)	2620-2448	P303/Page108/PF3340 /S135/Z765	G2a2b2a
MX299	Oberbipp Horgen (CH)	2910-2679	PF3147	G2a2a
MX304	Auvernier (CH)	2866-2601 date from individual of the same burial	CTS5330	R1b1a2a1a
RA58	Muttenz (CH)	2937-2886	FGC7739/Z6488	G2a2a1a2a
RA61	Muttenz (CH)	2905-2865	PF3239	G2a2a1a2a1
RA62	Muttenz (CH)	2921-2886	PF3239	G2a2a1a2a1
RA63	Zuzach (CH)	2046-1946	L2/S139	R1b1a1b1a1a2b1
RA64	Zuzach (CH)	2206-2126	L2/S139	R1b1a1b1a1a2b1
SX10	Rapperswill Zürichstrasse (CH)	new-born or unborn baby of SX8	L166	G2a2a1a2a1a
SX11	Niederried Ursisbalm (CH)	4458 - 4362	PF3239	G2a2b2a1a1
SX20	Wartau (CH)	1693-1609	P312/PF6547/S116	R1b1a1b1a1a2
SX29	Lingolsheim (F)	no collagen	M423	I2a1a2
SX32	Lingolsheim (F)	2463-2208	P310/PF6546/S129	R1b1a1b1a1
SX33	Lingolsheim (F)	4766-4601	L161.1/S185.1	I2a1a2a

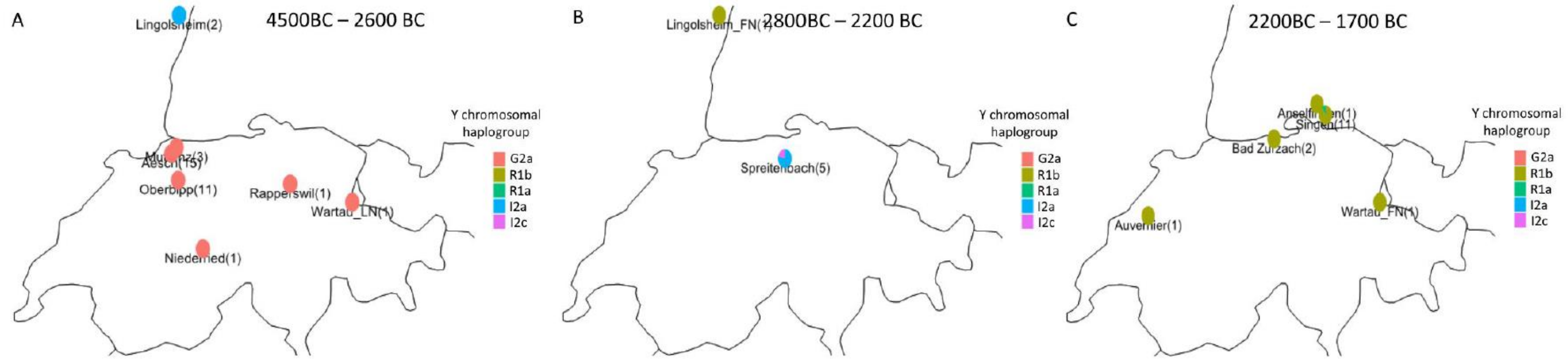

Supplementary Figure 2 | Composition of the Y chromosomal haplogroups for each site at different time spans. Map generated with R version 3.4.3 (R Core Team 2017) using the CIA World Data Bank II is currently (mid-2003) available from http://www.evl.uic.edu/pape/data/WDB/.

Supplementary Note 5: ADMIXTURE Analysis

A dataset of all newly sequenced ancient individuals from Switzerland and 275 already published ancient genomes as well as modern populations from the HO dataset were used for ADMIXTURE (version 1.3) analysis. Before starting the analysis, the dataset was pruned for LD using PLINK version v1.07 and the parameters --*indep-pairwise 200 25 0.5*.
The ADMIXTURE software was run in unsupervised mode and the optimal number of k was determined using ADMIXTURE's cross-validation procedure (SI Fig 3).

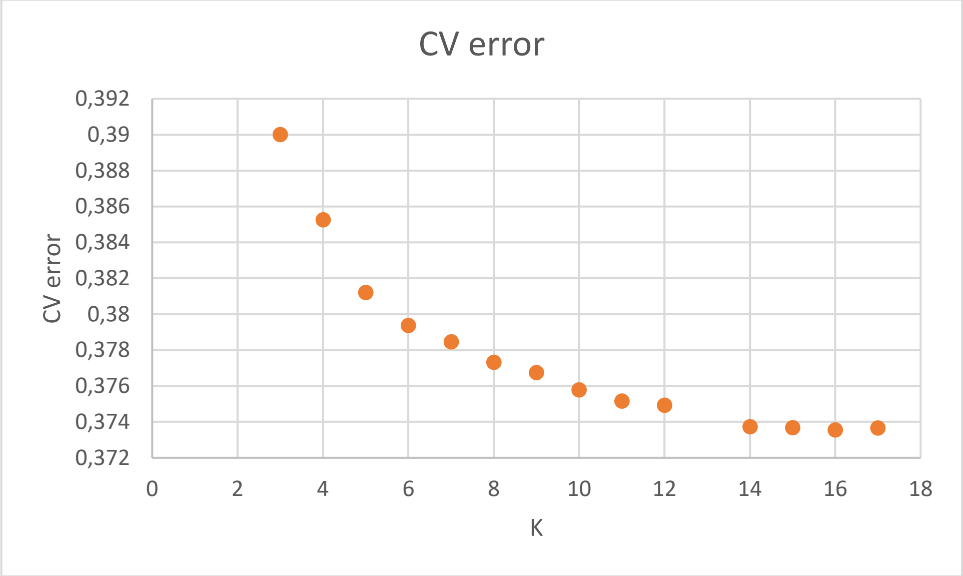

Supplementary Figure 3 | CV error for k between 3 and 17. The lowest CV value can be found for k=16.

As k=16 produced the lowest CV values, the results of this clustering were plotted (SI Fig 4). These results represent the individuals from sites older than 2700 BCE as a mixture of clusters being similar to the Earls Farmers from Anatolia and WHG. Individuals from sites younger than 2700 BCE also show one additional ancestry cluster which can also be found in Yamnaya individuals from the Pontic Steppe.

Supplementary Figure 4 | Unsupervised ADMIXTURE plot (k=16) of the newly sequenced ancient Swiss individuals and selected published ancient genomes. The clustering supports the results of the PCA plot, displaying the ancient Swiss individuals as a mixture of Anatolian Farmers and WHG for sites older than 2700 BCE and showing additional ancestry in the sites younger than 2700 BCE being similar to Yamnaya-like individuals from the Pontic Steppe.

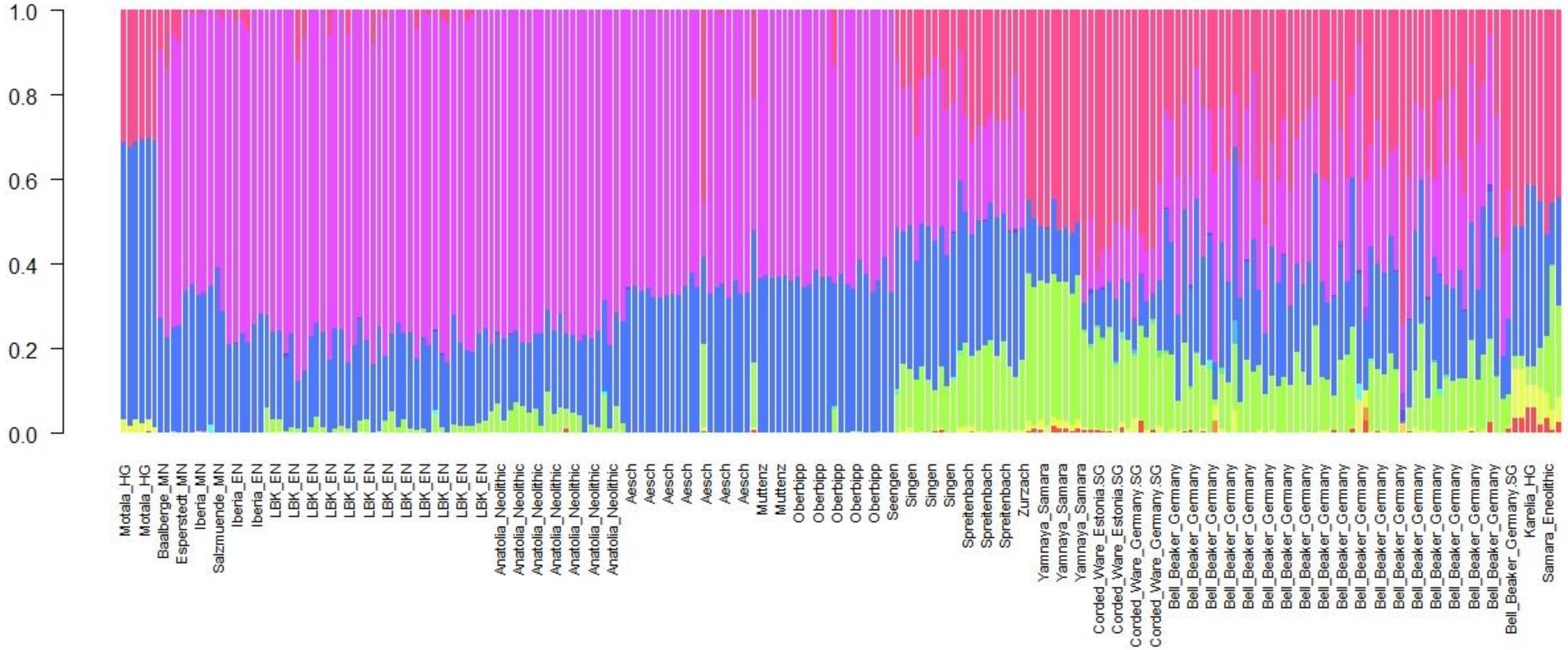

Supplementary Note 6: Sex-biased Admixture

To assess, if the admixture process between Late Neolithic individuals and immigrants with 'steppe'-related ancestry shows any sex-bias, f3 statistics of the form (Mbuti; Source; Test) using *qp3Pop* from ADMIXTOOLS (https://github.com/DReichLab) were performed on the autosomal SNPs of the HO panel and on 34,207 X chromosomal SNPs that represent the intersect of position on the X chromosome that are covered sufficiently in the individuals from the Final Neolithic sites: Singen, Spreitenbach, and Wartau_FN. As source Corded_Ware_Germany, Yamnaya_Samara, Globular_Amphora, and the Late Neolithic Swiss Sites were tested, similar to the approach described in (**Saag et al. 2017**).

The Late Neolithic individuals from our study (Spreitenbach, Singen, and Wartau_FN) are relatively symmetrically related to the sources Swiss_LN and Globluar_Amphora when comparing X chromosomes to autosomes. Relative to this the Late Neolithic Swiss individuals or our study have a higher affinity on the autosomes than on the X chromosome using the sources CWC and Yamnaya_Samara. The observed effect is stronger using Yamnaya_Samara as a source than when CWC is used. These results suggest that the influence of the steppe was stronger on the male side (SI Fig. 5). These results match the results of previously published data (**Saag et al. 2017)**.

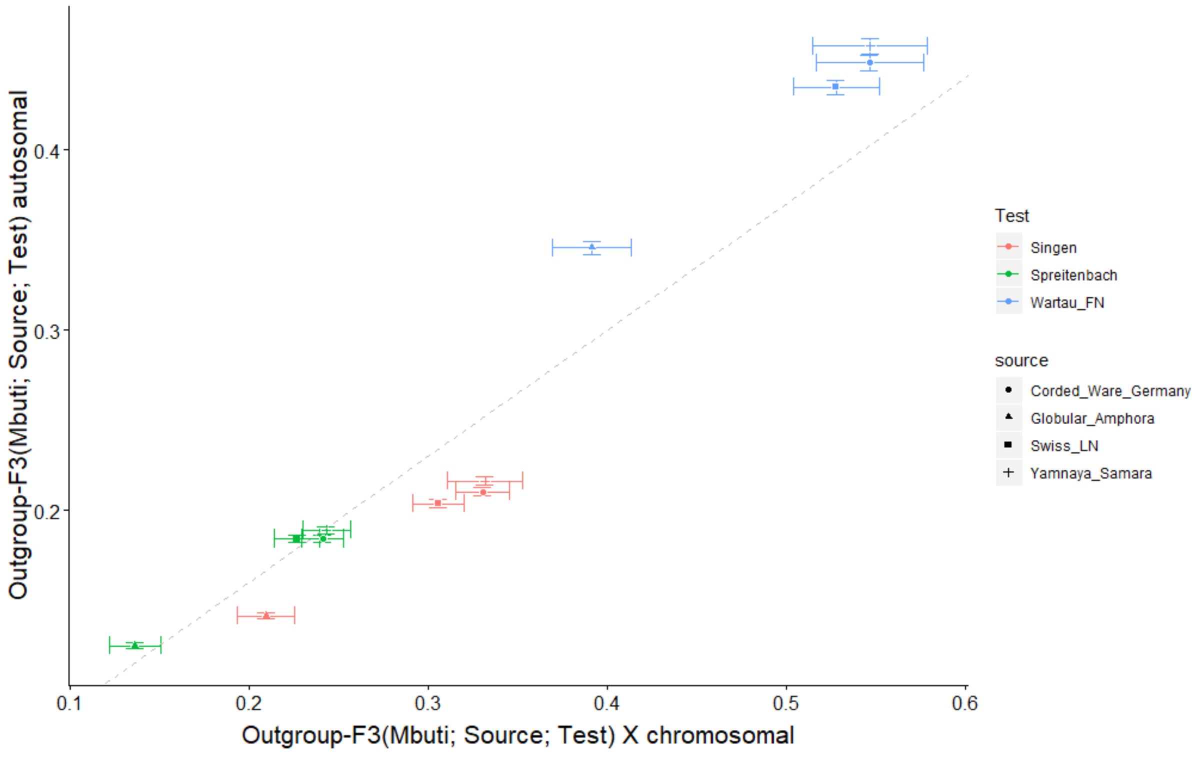

Supplementary Figure 5 | Sex-biased admixture patterns in Final Neolithic Swiss individuals. F 3 values of statistics of the form (Mbuti; Source; Test) for autosomes and the X chromosome. Error bars represent standard deviaition of the *f3* statistics.

Supplementary Note 7: Proportions of steppe ancestry on individual level

Relative proportions of ancestry components were estimated using *qpAdm* from ADMIXTOOLS (https://github.com/DReichLab).
Each individual's ancestry was modelled using a three-way model with the reference populations WHG, Anatolia Neolithic (ANF) and Yamnaya Samara (YAM). Subsequently, the best supported minimal model was selected and if necessary, thus for individuals for which a two-way model was better supported with either omitting ANF or WHG, the corresponding two-way model was rerun. In cases were also the two-way model was rejected Yamnaya proportions were set either to 0 or 1. Individuals with less than 100k SNPs were filtered out.
As seen in SI Figure 6 before 2800 BC the steppe component is absent in Central Europe. The earliest individuals with proportions of steppe ancestry greater than zero can be found in the Swiss dataset. However, the ancient Dataset from Great Britain and the MES region in Germany show larger gaps for this specific time span might also biasing the results.
In all regions, an increase of the steppe component around 2500 BC can be seen.

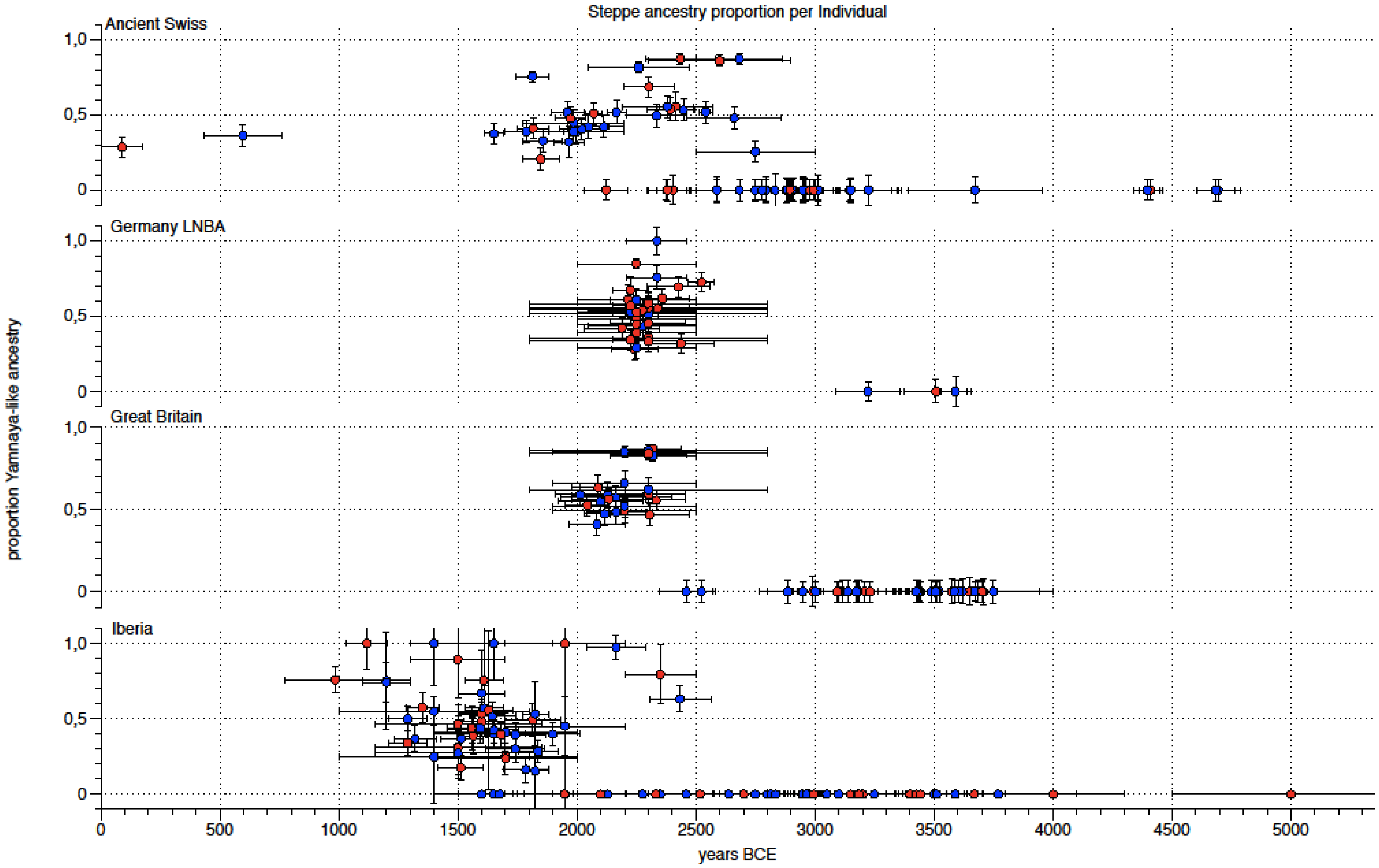

Supplementary Figure 6 | Relative proportions of steppe ancestry in each individual over time. We used *qpAdm* to estimate the proportion of steppe ancestry in each individual. Red dots female individuals and blue dots male individuals. Horizontal error bars represent ranges of C14 dates and vertical error bars represent standard errors of the steppe-related ancestry.

Supplementary Note 8: Estimated arrival time of the steppe component in the different regions

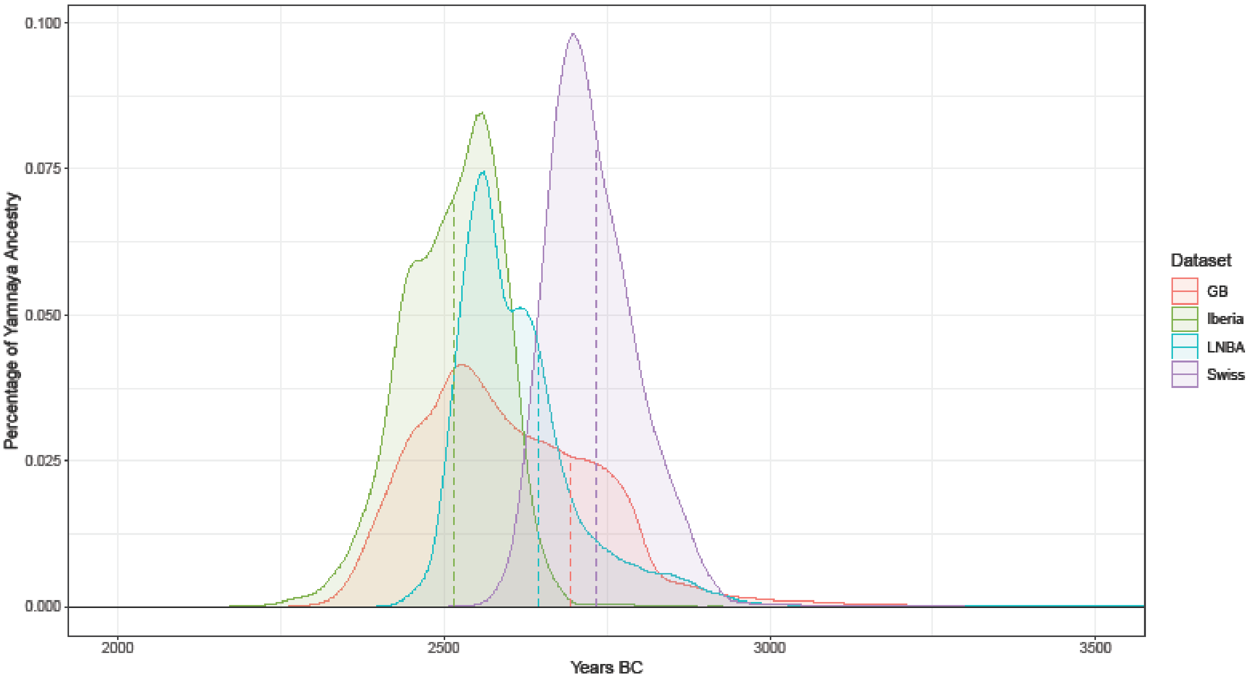

Supplementary Figure 7 | Density plots of the percentage of Yamnaya ancestry in each individual of the regions Great Britain, Iberia, Switzerland, and Germany. The earliest increase can be detected in Switzerland.

Supplementary Table 2 | qpAdm admixture models for the individuals of each site. P-values greater than 0.05 (model is not rejected) marked in green.

Test: WHG Anatolia_Neolithic Yamnaya_Samara
Using the right outgroups: Mbuti Ust_Ishim_HG_published.DG Ethiopia_4500BP.SG MA1_HG.SG Villabruna Papuan Onge Han Karitiana

Population	WHG	Anatolia N	Yamnaya	SD (WHG)	SD (Anatolia N)	SD (Yamnaya)	tail prob (000)	tail prob (001)	tail prob (010)	tail prob (100)
Bell_Beaker_Germany	0,16	0,40	0,44	0,01	0,02	0,02	0,022	0,000	0,000	0,000
Corded_Ware_Germany	0,11	0,22	0,66	0,17	0,04	0,04	0,348	0,000	0,000	0,000
LBK_EN	0,06	0,94	0,00	0,01	0,02	0,00	0,105	0,158	0,000	0,000
Auvernier	0,00	0,79	0,21	0,00	0,14	0,14	0,249	0,212	0,000	0,316
Burgäschisee	0,00	0,70	0,30	0,00	0,15	0,16	0,656	0,355	0,002	0,764
Spreitenbach	0,14	0,38	0,48	0,02	0,03	0,03	0,014	0,000	0,000	0,000
Anselfingen	0,15	0,32	0,54	0,04	0,09	0,09	0,022	0,000	0,000	0,000
Singen	0,19	0,42	0,39	0,02	0,03	0,03	0,126	0,000	0,000	0,000
Oberbipp_F	0,22	0,76	0,02	0,05	0,09	0,09	0,986	0,987	0,000	0,000
Lingolsheim_FN	0,14	0,39	0,47	0,04	0,08	0,08	0,274	0,000	0,000	0,000
Wartau_FN_1	0,17	0,44	0,39	0,02	0,06	0,06	0,621	0,000	0,000	0,000
Wartau_R	0,14	0,59	0,27	0,03	0,07	0,07	0,450	0,005	0,000	0,000
Zurzach	0,18	0,38	0,44	0,03	0,06	0,06	0,719	0,000	0,000	0,000
Lingolsheim_MN	0,20	0,80	0,00	0,02	0,05	0,00	0,209	0,275	0,00	0,000
Muttenz	0,27	0,72	0,00	0,02	0,04	0,00	infeasible	0,227	0,000	infeasible
Niederried	0,22	0,78	0,00	0,02	0,05	0,00	infeasible	0,249	0,000	infeasible
Oberbipp	0,28	0,73	0,00	0,02	0,03	0,00	infeasible	0,123	0,000	infeasible
Seengen	0,26	0,74	0,00	0,05	0,11	0,00	0,304	0,395	0,000	0,000
Rapperswil	0,21	0,79	0,00	0,03	0,06	0,00	infeasible	0,062	0,000	infeasible
Wartau_LN	0,20	0,80	0,00	0,03	0,07	0,00	infeasible	0,579	0,000	0,000
Wartau_FN_2	0,15	0,85	0,00	0,02	0,05	0,00	infeasible	0,200	0,000	infeasible
Aesch	0,26	0,74	0,00	0,01	0,03	0,03	0,135	0,162	0,000	0,000

Supplementary Table 3 | DATES results on individual level with Yamnaya_Samara and Swiss_LN as sources

Sample	Site	sex	maxageBP	minageBP	ageBP	mean generations	generations	gen +/-	maxgen	mingen
MX188	Spreitenbach	m	2495	2399	2447	22,91	19,13	11,29	30,42	7,84
MX190	Spreitenbach	m	2860	2460	2660	495,46	407,65	360,26	767,91	47,39
MX192	Spreitenbach	m	2571	2513	2542	186,36	130,21	75,12	205,32	55,09
MX195	Spreitenbach	m	2470	2050	2260	6,90	6,01	3,33	9,34	2,69
MX197	Spreitenbach	f	2490	2294	2392	14,56	14,89	2,14	17,03	12,75
MX198	Spreitenbach	f	2900	2300	2600	7,81	8,63	3,40	12,03	5,22
MX199	Spreitenbach	f	2409	2197	2303	9,38	6,79	7,41	14,19	-0,62
MX252	Singen	m	1941	1774	1858	111,38	82,75	62,19	144,94	20,56
MX254	Singen	m	2199	1774	1987	50,43	48,08	17,76	65,85	30,32
MX257	Singen	m	1879	1696	1788	54,99	50,92	14,58	65,50	36,34
MX259	Anselfingen	m	2456	2203	2330	10,10	8,54	7,62	16,16	0,92
MX270	Singen	m	2199	1774	1987	30,51	23,85	13,54	37,39	10,32
MX275	Singen	m	2135	1961	2048	39,76	36,18	18,40	54,58	17,78
MX279	Singen	m	1882	1745	1814	35,74	113,66	74,86	188,53	38,80
MX280	Singen	f	2035	1910	1973	35,84	31,70	9,83	41,53	21,87
MX283	Singen	m	2116	1926	2021	18,75	1,77	14,71	16,48	0,00
MX288	Singen	m	2199	2028	2114	68,01	43,70	39,60	83,30	4,10
RA63	Zurzach	m	2046	1946	1996	13,58	12,69	4,15	16,84	8,55
RA64	Zurzach	m	2206	2126	2166	16,54	16,90	4,41	21,31	12,49
SX20	Wartau	m	1693	1609	1651	17,96	15,25	6,00	21,25	9,25
SX23	Wartau	f	1883	1749	1816	16,48	13,18	7,22	20,41	5,96
SX26	Wartau		2461	2295		30,77	28,76	13,385	42,15	15,38
MX189	Spreitenbach	f	2105	2036	2071	243,16	wired number			
MX191	Spreitenbach	m	2570	2190	2380	437,29	wired number			
MX194	Spreitenbach	f	2489	2344	2417	304,99	wired number			
MX196	Spreitenbach	f	2580	2290	2435	5,71	wired number			
MX277	Singen	f	1926	1770	1848	34,16	wired number			
MX256	Singen	f	2132	1922	2027	0,00	wired number			
MX258	Singen	m	2028	1903	1966	28,90	wired number			

MX265	Singen	m	763	431	597	wired number	wired number				
MX298	Wartau	m	2620	2448	2534	wired number	wired number				
MX304	Auvernier	m	2866	2601	2734	208,80	wired number				
MX310	Burgaeschisee	m	2862	2581	2722	135,01	wired number				
MX286	Singen	m	2029	1892	1961	64,10	-271,57	237,27	-34,30	-508,85	
MX251	Singen	f	2197	1981	2089	5,12	-134,78	109,41	-25,38	-244,19	

Supplementary Table 4 | DATES results for different regions

Region	Group	Source1	Source2	C14 range max	C14 range min	generations	gen +/-
Swiss	Spreitenbach_CWC	Yamnaya_Samara	Swiss_LN	2900	2036	10,934	1,78
Swiss	Swiss_EBA	Yamnaya_Samara	Swiss_LN	2456	1609	27,89	3,757
MES	Bell_Beaker_Germany	Yamnaya_Samara	MES_Neolithic	2625	2050	34,817	11,611
Iberia	Bell_Beaker_Iberia	Yamnaya_Samara	Iberia_MN	2800	1776	7,258	7,183
GB	Bell_Beaker_England	Yamnaya_Samara	England_Neolithic	2800	1800	22,114	2,482
MES	Corded_Ware_Germany	Yamnaya_Samara	MES_Neolithic	2578	2033	26,722	9,641

Supplementary Table 5 | Relatedness estimates using PMR, READ and lcMLkin for first and second degree related individuals

		lcMLkin						READ			PMR				
Ind1	Ind2	k0_hat	k1_hat	k2_hat	pi_HAT	nbSNP	r	Relationship	Z_upper	Z_lower	nSNPs	nmismatch	pmismatch	same MT DANN	same Y HG
MX270	MX275	0,315	0,461	0,223	0,454	167647	0,4535	First Degree	7,21	-14,07	196616	38132	0.19394	yes	no
MX209	MX219	0,231	0,639	0,13	0,45	24866	0,4495	First Degree	8,42	-3,97	29551	5458	0.18470	no	no
SX10	SX8	0,13	0,847	0,023	0,446	124180	0,4465	First Degree	9,44	-13,34	145624	28231	0.19386	yes	na
MX195	MX197	0,119	0,873	0,008	0,445	294763	0,4445	First Degree	3,92	-20,07	356136	69688	0.19568	yes	na
MX187	MX212	0,13	0,852	0,018	0,444	175276	0,444	First Degree	8,39	-12,11	208924	39351	0.18835	no	no
MX192	MX197	0,12	0,873	0,007	0,443	285900	0,4435	First Degree	4,75	-20,44	344641	67734	0.19653	yes	na
MX192	MX195	0,334	0,476	0,19	0,428	268894	0,428	First Degree	1,96	-17,57	323615	63541	0.19635	yes	no
MX188	MX190	0,382	0,39	0,227	0,422	326168	0,422	First Degree	2,89	-18,45	394714	78180	0.19807	yes	no
MX254	MX286	0,166	0,828	0,006	0,42	329141	0,42	First Degree	4,23	-23,34	396702	78410	0.19765	no	no
MX283	MX286	0,185	0,807	0,007	0,411	340960	0,4105	First Degree	5,46	-24,03	411459	81328	0.19766	no	no
MX275	MX286	0,204	0,784	0,013	0,405	244748	0,405	First Degree	3,41	-23,07	291501	57925	0.19871	no	no
MX150	MX187	0,248	0,71	0,042	0,397	91284	0,397	First Degree	8,22	-12,18	109655	20743	0.18917	no	no
MX254	MX283	0,439	0,422	0,138	0,35	393973	0,349	First Degree	6,13	-16,49	477609	97660	0.20448	yes	yes
MX183	MX211	0,422	0,47	0,108	0,343	258399	0,343	First Degree	0,69	-19,35	310268	62928	0.20282	yes	no
MX150	MX209	0,577	0,346	0,077	0,25	72301	0,25	Second Degree	8,62	-0,25	86029	18175	0.21127	no	no
MX275	MX283	0,577	0,407	0,016	0,22	286647	0,2195	Second Degree	5,68	-10,40	343024	75468	0.22001	no	yes
MX270	MX286	0,614	0,357	0,028	0,207	189791	0,2065	Second Degree	3,75	-11,33	223865	50077	0.22369	no	no
MX254	MX275	0,679	0,3	0,021	0,171	277631	0,171	Second Degree	2,92	-13,44	331845	75516	0.22756	no	yes
MX187	MX219	0,754	0,193	0,054	0,15	30547	0,1505	Second Degree	2,92	-3,09	36392	8053	0.22128	no	no
MX187	MX209	0,705	0,291	0,003	0,149	181634	0,1485	Second Degree	2,51	-9,68	216996	48874	0.22523	yes	no
MX150	MX219	0,764	0,223	0,013	0,125	12304	0,1245	Second Degree	1,62	-2,35	14706	3285	0.22338	no	no

MX150	MX212	0,82	0,121	0,059	0,12	70041	0,1195	Second Degree	1,76	-6,66	83579	18906	0.22621	no	no
Aes3	Aes12	0,123	0,863	0,014	0,445	279742	0,4455	First Degree	12,41	-16,25	331487	63428	0.19134	yes	na
Aes3	Aes24	0,583	0,41	0,008	0,213	325204	0,213	Second Degree	7,56	-9,51	386396	84889	0.21969	no	na
Aes12	Aes24	0,143	0,847	0,009	0,433	273774	0,4325	First Degree	7,14	-19,38	324166	62519	0.19286	no	na
Aes3	Aes19	0,104	0,891	0,005	0,45	323734	0,4505	First Degree	12,20	-16,34	384537	73727	0.19173	yes	na
Aes19	Aes24	0,577	0,409	0,013	0,218	316956	0,2175	Second Degree	7,21	-9,06	376691	82322	0.21854	no	na
Aes11	Aes17	0,106	0,878	0,015	0,454	308832	0,454	First Degree	11,54	-16,95	366976	70202	0.1913	no	na
Aes14	Aes15	0,106	0,878	0,015	0,454	193594	0,454	First Degree	12,84	-14,64	227595	43354	0.19049	yes	na
Aes12	Aes19	0,354	0,437	0,208	0,427	272655	0,4265	First Degree	10,74	-12,73	322847	61942	0.19186	yes	na

Supplementary Table 6 | D-statistics.

Site	lat	lon	steppe_ancestry	PopA	PopB	F4	Z	ka	ka1	ka2
Spreitenbach	47,255	8,2158	yes	Swiss-French	Swiss-German	0,000114	2,056	6487	6476	98548
Singen	47,767097	8,872239	yes	Swiss-French	Swiss-German	0,000053	0,865	5911	5906	89516
Wartau_FN_1	47,45	9,292	yes	Swiss-French	Swiss-German	-0,000015	-0,196	4775	4776	72386
Wartau_FN_2	47,45	9,292	yes	Swiss-French	Swiss-German	-0,000025	-0,336	4618	4619	70418
Wartau_LN	47,45	9,292	no	Swiss-French	Swiss-German	-0,000009	-0,09	3387	3387	51365
Lingolsheim_FN	48,554457	7,681749	yes	Swiss-French	Swiss-German	0,000082	0,711	2262	2259	34356
Anselfingen	47,854144	8,7737404	yes	Swiss-French	Swiss-German	0,00018	1,371	1754	1750	26337
Zurzach	47,587789	8,293325	yes	Swiss-French	Swiss-German	0,000099	1,207	4199	4193	63804
Burgaeschisee	47,101	7,406	yes	Swiss-French	Swiss-German	-0,000355	-1,425	307	309	4760
Auvernier	46,5835	6,5245	yes	Swiss-French	Swiss-German	0,000193	0,763	330	329	5038
Oberbipp	47,262067	7,659835	no	Swiss-French	Swiss-German	-0,000057	-0,967	6387	6393	96637
Muttenz	47,3122	7,3843	no	Swiss-French	Swiss-German	0,000051	0,767	5401	5397	81812
Seengen	47,1936	8,122	no	Swiss-French	Swiss-German	-0,000435	-2,626	821	826	12666
Niederried	47,0117074	7,2505417	no	Swiss-French	Swiss-German	-0,000198	-2,57	5107	5122	77394
Rapperswil	47,2266239	8,8184374	no	Swiss-French	Swiss-German	0,000079	0,814	2948	2944	44774
Aesch	47,465413	7,601691	no	Swiss-French	Swiss-German	-0,000063	-1,138	6721	6767	102298
Lingolsheim	48,554457	7,681749	no	Swiss-French	Swiss-German	0,000018	0,257	4622	4620	70266
Spreitenbach	47,255	8,2158	yes	Swiss-Italian	Swiss-German	-0,000519	3,573	6516	6465	98548
Singen	47,767097	8,872239	yes	Swiss-Italian	Swiss-German	-0,000534	3,665	5940	5893	89516
Wartau_FN_1	47,45	9,292	yes	Swiss-Italian	Swiss-German	-0,000516	2,736	4798	4760	72386
Wartau_FN_2	47,45	9,292	yes	Swiss-Italian	Swiss-German	-0,000042	0,239	4629	4627	70418
Wartau_LN	47,45	9,292	no	Swiss-Italian	Swiss-German	-0,000233	1,023	3397	3385	51365
Lingolsheim_FN	48,554457	7,681749	yes	Swiss-Italian	Swiss-German	-0,001037	3,866	2280	2244	34356
Anselfingen	47,854144	8,7737404	yes	Swiss-Italian	Swiss-German	-0,000695	2,09	1764	1746	26337
Zurzach	47,587789	8,293325	yes	Swiss-Italian	Swiss-German	-0,000688	3,407	4223	4179	63804
Burgaeschisee	47,101	7,406	yes	Swiss-Italian	Swiss-German	-0,000594	0,93	311	308	4760
Auvernier	46,5835	6,5245	yes	Swiss-Italian	Swiss-German	-0,000898	1,316	334	329	5038
Oberbipp	47,262067	7,659835	no	Swiss-Italian	Swiss-German	0,000046	-0,321	6401	6405	96637

Muttenz	47,3122	7,3843	no	Swiss-Italian	Swiss-German	-0,000092	0,561	5413	5405	81812
Seengen	47,1936	8,122	no	Swiss-Italian	Swiss-German	-0,000069	0,169	826	825	12666
Niederried	47,0117074	7,2505417	no	Swiss-Italian	Swiss-German	0,000068	-0,371	5121	5126	77394
Rapperswil	47,2266239	8,8184374	no	Swiss-Italian	Swiss-German	-0,000389	1,64	2959	2942	44774
Aesch	47,465413	7,601691	no	Swiss-Italian	Swiss-German	-0,00003	0,026	6738	6738	101710
Lingolsheim	48,554457	7,681749	no	Swiss-Italian	Swiss-German	0,000099	-0,534	4627	4633	70266
Spreitenbach	47,255	8,2158	yes	Swiss-French	Swiss-Italian	-0,000405	-2,978	6473	6513	98548
Singen	47,767097	8,872239	yes	Swiss-French	Swiss-Italian	-0,000481	-3,431	5897	5940	89516
Wartau_FN_1	47,45	9,292	yes	Swiss-French	Swiss-Italian	-0,000531	-3,006	4762	4801	72386
Wartau_FN_2	47,45	9,292	yes	Swiss-French	Swiss-Italian	-0,000067	-0,399	4629	4633	70418
Wartau_LN	47,45	9,292	no	Swiss-French	Swiss-Italian	-0,000241	-1,126	3386	3399	51365
Lingolsheim_FN	48,554457	7,681749	yes	Swiss-French	Swiss-Italian	-0,000956	-3,897	2246	2279	34356
Anselfingen	47,854144	8,7737404	yes	Swiss-French	Swiss-Italian	-0,000515	-1,649	1749	1762	26337
Zurzach	47,587789	8,293325	yes	Swiss-French	Swiss-Italian	-0,000589	-3,068	4183	4221	63804
Burgaeschisee	47,101	7,406	yes	Swiss-French	Swiss-Italian	-0,000949	-1,648	307	311	4760
Auvernier	46,5835	6,5245	yes	Swiss-French	Swiss-Italian	-0,000705	-1,099	329	333	5038
Oberbipp	47,262067	7,659835	no	Swiss-French	Swiss-Italian	-0,000011	-0,079	6406	6407	96637
Muttenz	47,3122	7,3843	no	Swiss-French	Swiss-Italian	-0,000041	-0,255	5409	5412	81812
Seengen	47,1936	8,122	no	Swiss-French	Swiss-Italian	-0,000504	-1,273	823	829	12666
Niederried	47,0117074	7,2505417	no	Swiss-French	Swiss-Italian	-0,000131	-0,75	5123	5133	77394
Rapperswil	47,2266239	8,8184374	no	Swiss-French	Swiss-Italian	-0,00031	-1,389	2946	2959	44774
Aesch	47,465413	7,601691	no	Swiss-French	Swiss-Italian	-0,000066	-0,52	6738	6745	101710
Lingolsheim	48,554457	7,681749	no	Swiss-French	Swiss-Italian	0,000117	0,64	4636	4628	70266

References

1. Tschumi, O. Die steinzeitlichen Hockergräber der Schweiz. Anzeiger für schweizerische Altertumskunde : Neue Folge Indicateur d'antiquités suisses : Nouvelle série 22 (1920)
2. Hug E. 1956. Die Anthropologische Sammlung im Naturhistorischen Museum Bern. In: Bern NM, editor. Bern: Naturhistorisches Museum Bern.
3. Schlaginhaufen O. 1924b. Das Hockerskelett von Ursisbalm bei Niederried (Kt. Bern). Jahrbuch des Bernischen Historischen Museums in Bern:96-109.
4. Lichardus-Itten, M. Die Gräberfelder der Grossgartacher Gruppe im Elsaß (Habelt, Bonn, 1980)
5. Ramstein, M., Schimmelpfennig, D., Lösch, S. Ein neolithischer Dolmen an der Steingasse in Oberbipp. Archäologie Schweiz 37(3), 4-15 (2014).
6. Siebke, I. et al. The application of different 3D-scan-systems and photogrammetry at an excavation — A Neolithic dolmen from Switzerland. Digital Applications in Archaeology and Cultural Heritage 10, e00078; 10.1016/j.daach.2018.e00078 (2018)
7. Siebke I, Steuri N, Furtwängler A, Ramstein M, Arenz G, Hafner A, Krause J, and Lösch S. 2019. Who lived on the Swiss Plateau around 3300 BCE? - Analyses of commingled Human Skeletal Remains from the Dolmen of Oberbipp. International Journal of Osteoarchaeology. DOI: 10.1002/oa.2791
8. Bleuer E, Doppler T, Fetz H.. Gräber im näheren und weiteren Umfeld von Spreitenbach in Spreitenbach-Moosweg (Aargau, Schweiz): Ein Kollektivgrab um 2500 v Chr (ed. Doppler, T.) 233-266 (Basel 2012)
9. Löhlein W. Monumentale Grabanlagen der Steinzeit Megalithische Denkmale der Hochrheinregion. Denkmalpflege in Baden-Württemberg 3 2011
10. Stöckli WE, Niffeler U, and Gross-Klee E, editors. 1995. Neolithikum - Néolithique - Neolitico. SPM II. Basel. 385 p.
11. Schwegler U. 2016. Chronologie und Regionalität neolithischer Kollektivgräber in Europa und in der Schweiz Hochwald (Schweiz): LIBRUM Publishers & Editors LLC.
12. Bay R. 1936. Kiefer und Zähne aus dem neolithischen Steinkistengrab bei Aesch. Tätigkeitsbericht der Naturforschenden Gesellschaft Baselland 11 (1936-1938):22-30.
13. Doppler, T. Spreitenbach-Moosweg: ein Kollektivgrab um 2500 v. Chr. Archäologie Schweiz, Vol. 51 (2012).
14. Doswald C. Kaufmann B. Scheidegger S. Ein neolithisches Doppelhockergrab in Zurzach. Archäologie der Schweiz 12 (1989)
15. Grüninger I. and Kaufmann B. Ein Steinkistengrab von Rapperswil SG. Archäologie der Schweiz 5 (1982)
16. Stehrenberger T. Obere Höhle bei der "Procha Burg" Höhlenpost 148 (2016).
17. Stehrenberger T, Siebke I, and Lösch S. 2019. Sekundär bestattet oder «entsorgt»? Die Toten aus der Oberen Höhle Procha Burg (Wartau, Gretschins SG). In: Schweizerische Gesellschaft für Höhlenforschung, editor. Sinterlaken - Nationaler Kongress für Höhlenforschung Interlaken: accepted.
18. Stockhammer, P. W., Massy, K., Knipper, C., Friedrich, R., Kromer, B., Lindauer, S., et al. Rewriting the central European early bronze age chronology: Evidence from large-scale radiocarbon dating. PLoS One, 10(10), e0139705 (2015)

19. OELZE, V. M., NEHLICH, O. & RICHARDS, M. P. ‘THERE'S NO PLACE LIKE HOME’-NO ISOTOPIC EVIDENCE FOR MOBILITY AT THE EARLY BRONZE AGE CEMETERY OF SINGEN, GERMANY. Archaeometry 54, 752–778; 10.1111/j.1475-4754.2011.00644.x (2012).
20. R. Krause, Die endneolithischen und frühbronzezeitlichen Grabfunde auf der Nordstadtterrasse von Singen am Hohentwiel. Forschungen und Berichte zur Vor- und Frühgeschichte in Baden-Württemberg 32 (Stuttgart 1988).
21. Matthias B. Merkl CORDED WARE, BELL BEAKERS AND THE EARLIEST BRONZE AGE IN THE HEGAU AND THE WESTERN LAKE CONSTANCE REGION MUSAICA ARCHAEOLOGICA 1 (2016)
22. Ulrich-Bochsler S, Cooper C, Meyer L, Rüttimann D, and Lanz C. 2012. Anthropologische Bearbeitung der Skelettreste vom Burgäschisee. In: Wey O, editor. Die Cortaillod-Kultur am Burgäschisee Materialvorlage und Synthese zu den neolithischen Fundkomplexen von Burgäschisee-Ost, -Südwest, -Süd und -Nord. Bern: Stämpfli Verlag AG. p 111-154.
23. Schlaginhaufen O. 1924a. Die anthropologischen Funde aus den Pfahlbauten der Schweiz. Pfahlbauten, zehnter Bericht. Mitteilungen der Anthropologischen Gesellschaft in Zürich 29(4):220-241.
24. Wiedmer-Stern J. 1904. Archäologie aus dem Oberaargau. Archiv des Historischen Vereins des Kantons Bern 17 (1903-1904)(2).
25. Steuri, N. et al. MULTIPLE RADIOCARBON DATING OF HUMAN REMAINS: CLARIFYING THE CHRONOLOGY AND SEQUENCES OF BURIALS IN THE LATE NEOLITHIC DOLMEN OF OBERBIPP (SWITZERLAND). Radiocarbon II, 1–13; 10.1017/RDC.2019.96 (2019).
26. Siebke I. et al. Crops vs. Animals: Regional Differences in Subsistence Strategies of Swiss Neolithic Farmers revealed by Stable Isotopes. *Submitted* (2019).
27. Furtwängler, Anja, et al. "Ratio of mitochondrial to nuclear DNA affects contamination estimates in ancient DNA analysis." *Scientific reports* **8.1** (2018): 14075.
28. Peltzer, Alexander, et al. "EAGER: efficient ancient genome reconstruction." *Genome biology* **17.1** (2016): 60.
29. Renaud, Gabriel, et al. "Schmutzi: estimation of contamination and endogenous mitochondrial consensus calling for ancient DNA." *Genome biology* **16.1** (2015): 224.
30. Weissensteiner, H. et al. HaploGrep 2: mitochondrial haplogroup classification in the era of high-throughput sequencing. *Nucleic Acids Research* **44**, W58-63 (2016).
31. Saag, L. *et al.* Extensive Farming in Estonia Started through a Sex-Biased Migration from the Steppe. *Current biology : CB* **27,** 2185-2193.e6; 10.1016/j.cub.2017.06.022 (2017).
