# Supplementary material for: Ancient genomes reveal social and genetic structure of Late Neolithic Switzerland
Source: Nat Commun. 2020 Apr 20;11:1915. doi: 10.1038/s41467-020-15560-x (PMC7171184; doi:10.1038/s41467-020-15560-x)
Supplement: Supplementary file 7 — Description of Additional Supplementary Files [file 41467_2020_15560_MOESM7_ESM.pdf]

Title: Supplementary Data 1

Description: Summary of archaeological and genetic information for the 96 reported in this study

Title: Supplementary Data 2

Description: Summary of basic sequencing results of the 96 individuals reported in this study

Title: Supplementary Data 3

Description: Information on 399 previously published ancient individuals that were used for the population genetic analysis

Title: Supplementary Data 4

Description: qpAdm admixture models for each individual. P-values greater than 0.05 (model is not rejected) marked in green.
